# Analytical dilemmas in assessing efficacy of radical cure of vivax malaria

**DOI:** 10.1016/j.lansea.2026.100867

**Published:** 2026-09-10

**Authors:** J Kevin Baird

**Affiliations:** a Faculty of Medicine Universitas Indonesia, University of Oxford Clinical Research Unit Indonesia, Jakarta, Indonesia; b Nuffield Department of Medicine, Center for Tropical Medicine and Global Health, University of Oxford, Oxford, United Kingdom

The radical cure for *Plasmodium vivax* malaria involves administration of blood schizontocidal drug(s) against the acute attack and a hypnozoitocidal drug for the prevention of relapse due to latent hepatic stages of the parasite.^1^ There are many options for the former but just two for the latter: primaquine given daily for 7 or 14 days; or a single dose of tafenoquine. Currently the malaria control programme in India recommends 14 days of daily primaquine for radical cure of *P. vivax*.

In this issue of *The Lancet Regional Health Southeast Asia,* Baharia and colleagues have published a randomised controlled trial on efficacy of 7-day versus 14-day primaquine regimens of the same total dose for radical cure of *P. vivax* malaria in india.^2^ The findings align with other published evidence regarding these two regimens, i.e., equity in safety, tolerability, and efficacy.^3^ The authors acknowledge the pitfalls of not including a placebo control and a limited follow up duration of just 6 months. Depending on locality, Indian strains of *P. vivax* either relapse predominantly within 5 months or do so later than 6 months, corresponding to tropical versus more seasonal environments, respectively.^4^ The extremely low rates of relapse reported in the study in both treatment groups may indeed indicate the expected equity in efficacy, however the true rate of relapse at 6 months and beyond cannot be known with certainty. The study findings show that administering either regimen was associated with at least 6 months of nearly complete freedom from recurrent attacks by *P. vivax* and halving the dosing duration to 7 days would likely yield superior effectiveness through improved adherence. Several analytical considerations should be taken into account when interpreting these findings.

Primaquine therapy is influenced by several inherent patient level genetic characteristics prevalent in malaria endemic countries. It is a 8-aminoquinoline compound that may be toxic in patients with inherited glucose-6-phosphate dehydrogenase (G6PD) deficiency, which happens to be the most common inborn abnormality in endemic regions.^5^ The therapeutically active metabolite of primaquine is derived from the naturally variable cytochrome P450 isotype 2D6 (CYP2D6) that could include impaired phenotypes directly impacting therapeutic efficacy.^6^ The most common impaired CYP2D6 allele called ∗10 is reported to occur at high frequencies among Asian populations.^7^ Finally, the combined therapies of radical cure are known to pharmacologically interact in ways that makes primaquine either more or less efficacious.^8^

Another key analytical consideration is the geographically varied relapse behaviours of *P. vivax*.^9^ Some strains may relapse quickly and frequently, whereas others may either relapse months later or not at all. These relapse patterns invariably reflect the seasonal presence of the anopheline mosquito vectors.^10^ In any setting, the relapses continue to occur alongside infections borne of recent anopheline biting activity. Distinguishing such events by genetic analyses is made difficult by the intrinsic genetic heterogeneity of the latent hepatic reservoir. Although variable, relapse behaviours are often predictable by region and known temporal patterns therein. Consideration of these relapse patterns should guide decisions regarding appropriate periods of post-radical cure follow up.

There is an ethical dilemma regarding use of placebo control in assessing anti-relapse therapy. Some may argue that withholding treatment exposes participants to avoidable recurrent attacks. We must weigh that view against that of administering treatment not actually known to effectively avoid those attacks. In vivax malaria chemotherapeutics, such uncertainty is often the rule due to the logistical and financial challenges of conducting an appropriate placebo-controlled randomised trial for radical cure. Further and perhaps most importantly, research participants may ethically be asked to endure a certain degree of illness provided these conditions are met: the discomfort is neither severe, prolonged, or unrelievable; there is minimal and wholly minimised risk of death or permanent disability; there is no alternative means of answering the scientific question posed; the answer to that question is of an importance that merits the risks and discomforts imposed; and fully informed consent is freely provided. The now common practice of conducting controlled human malaria infection studies^11^ is no different in a clinical and ethical sense than a placebo control in a trial of radical cure, provided the clinical supervision is as rigorous. There is no compelling ethical reasoning to exclude a placebo control in these trials, while there are compelling reasons to apply it.^12^

While the trial of Baharia and colleagues offers preliminary evidence which may well be considered to guide policy on the radical cure of vivax malaria in India, definitive evidence of anti-relapse efficacy may require relapse control arms^13^^,^^14^ or certain knowledge of local relapse behaviours alongside defined reinfection rates within follow up periods tailored to those conditions. Ascertaining the efficacy of anti-relapse therapies remains one of the most technically challenging problems in malaria chemotherapeutics.

## Declaration of interests

The author declares no conflicts of interest.
